# A Gut Gone to Pot: A Case of Cannabinoid Hyperemesis Syndrome due to K2, a Synthetic Cannabinoid

**DOI:** 10.1155/2014/167098

**Published:** 2014-04-29

**Authors:** Anene Ukaigwe, Paras Karmacharya, Anthony Donato

**Affiliations:** The Reading Health System, 6th Avenue and Spruce Street, West Reading, PA 19611, USA

## Abstract

Cannabinoid Hyperemesis Syndrome (CHS) was first described in 2004. Due to its novelty, CHS is often unrecognized by clinicians leading to expensive workup of these patients with cyclical symptoms. It may take up to 9 years to diagnose CHS. CHS is characterized by cyclical nausea and vomiting, abdominal pain, and an unusual compulsion to take hot showers in the presence of chronic use of cannabinoids. Cannabicyclohexanol is a synthetic cannabinoid, popularly known as K2 spice. It is a popular marijuana alternative among teenagers and young adults since it is readily available as herbal incense. Unlike marijuana, many users know that K2 is not detected in conventional urine drug screens, allowing those users to conceal their intake from typical detection methods. Serum or urine gas chromatography mass spectrophotometry is diagnostic, though not widely available. Thus, it is imperative for clinicians to recognize CHS, even with negative UDS, to provide cost-effective care. We present a 38-year-old man with a 10-year history of cannabis, and 1-year history of K2 abuse admitted with 1-week history of episodes of nausea, vomiting of clear fluids, and epigastric discomfort. Symptoms are relieved only by hot showers. Extensive laboratory, radiologic, and endoscopic evaluation was unrevealing. CHS was diagnosed, based on proposed criteria by Simonetti et al.

## 1. Introduction

Although the well-documented antiemetic properties of cannabis account for its use in chemotherapy-induced nausea and vomiting, interestingly cannabis and related substances may induce vomiting as in Cannabinoid Hyperemesis Syndrome (CHS). The broad differentials of abdominal pain and vomiting and the relative novelty of cannabinoid hyperemesis condition can lead to expensive and unnecessary investigations. Diagnosis can be a challenge especially in patients using synthetic cannabinoids like K2 which are not detected in conventional urine drug screens. This case illustrates CHS with the use of the synthetic cannabinoid, K2, which is less commonly reported [1].

## 2. Background

Cannabinoids are the commonest illicit drug encountered worldwide [2]. The prevalence is estimated at 3 million cases and has increased in the past decade [3]. Although cannabis is more commonly associated with antiemetic properties, a paradoxical effect that results in cyclic nausea and vomiting has been reported. This paradoxical effect, called Cannabinoid Hyperemesis Syndrome, was first described by Allen et al. in 2004. It consists of cyclical abdominal pain, nausea, and vomiting in the setting of chronic cannabinoid abuse. An unusual and unique feature of this disorder is a strong compulsion to bathe or shower in hot water multiple times per day. Recognition of this syndrome is important in order to avoid costly and unnecessary evaluation. Recognizing this presentation also helps the patient understand and break the cycle of drug use.

## 3. Case Presentation

A 38-year-old man with a self-reported long-term history of standard cannabinoid use and K2 (a synthetic cannabis) presented to the emergency room with a 2-week history of nausea, vomiting, and severe abdominal pain. He smoked 3-4 times a day, occasionally up to 10 rolls a day with his last K2 use being the night before admission. He denied any other illicit drug use. He reported having similar episodes of nausea and vomiting in the past, which lasted 2-3 days and terminated without medical intervention. Past medical history was significant for nonulcer dyspepsia for which he took nonprescription antacids.

On physical examination, he was afebrile with temperature of 36.8°C, pulse of 89/min, a respiratory rate of 16/min, a blood pressure of 115/73 mmHg, and oxygen saturation of 98% while breathing ambient air. He appeared to be uncomfortable and was curled up in bed holding his abdomen. He was alert, active, and oriented to time, place, and person. He exhibited no nystagmus and had a nonfocal neurological exam. His abdomen was soft but exhibited tenderness in the epigastric and periumbilical regions with decreased bowel sounds but no rebound tenderness or guarding. He noted relief only with hot showering and, on follow-up, would often refuse to come out of the shower to meet the physicians.

Laboratory studies showed mild leukocytosis with a WBC count of 14 × 10^9^/L (normal: 4–11 × 10^9^/L). Serum electrolyte testing revealed a sodium of 123 mmol/L (normal: 135–153 mmol/L), potassium of 3.4 mmol/L (normal: 3.5–5.3 mmol/L), chloride of 74 mmol/L (normal: 98–109 mmol/L), and bicarbonate of 21 mmol/L (normal: 24–31 mol/L). Blood urea nitrogen was 160 mg/dL (normal: 5–26 mg/dL) and creatinine was 4.78 mg/dL (normal: 0.5–1.5 mg/dL). No baseline studies were available for comparison. Amylase, lipase, and liver function tests were normal. Plain abdominal imaging did not reveal any obstruction. Esophagogastroduodenoscopy (EGD) showed mild gastritis with unrevealing histology and microbiology. Conventional urine drug screen, which does not test for synthetic cannabinoids, was negative. His urinalysis was normal except for the presence of hyaline casts. His fractional excretion of sodium (FeNa) was 0.4 and urine sodium level was 10 mmol/L (normal: 10–60 mmol/L). Renal ultrasound was normal. Based on proposed diagnostic criteria for CHS, his self-reported synthetic cannabis use, and cyclic vomiting with negative organic evaluation, diagnosis of Cannabinoid Hyperemesis Syndrome with prerenal acute kidney injury was made. He was given intravenous fluids for his hypovolemia. Ondansetron was given for his nausea and vomiting, which offered no relief. His leukocytosis, nausea, and renal failure completely resolved 72 hours after admission; at that time his serum creatinine was 1.1 mg/dL. He was doing well and stayed off the K2 at outpatient follow-up 2 weeks later.

## 4. Discussion

Cannabinoid Hyperemesis Syndrome (CHS) is characterized by cyclic vomiting, abdominal pain, and compulsive need to take hot showers [4]. The frequent showers are believed to be a learned behavior, providing temporary relief of the symptoms for unclear reasons. It is an episodic, recurrent disorder with symptom free periods in-between. CHS may be divided into periods of prodrome, hyperemesis, and recovery [5]. The prodrome can last for months to years and is characterized by early morning nausea, abdominal discomfort, and fear of vomiting. This may lead to escalating use of cannabis for self-treatment of nausea. The hyperemetic phase is the classic CHS. This is characterized by frequent paroxysmal nausea and vomiting which may be enough to lead to disruption in social and occupational functioning [6]. The associated abdominal pain is usually diffuse, mild, and poorly characterized. It is in the hyperemetic phase that patients prefer frequent hot showers. Hence, it has also been called hyperemetic, hydrophilic syndrome. It is also during this phase that they frequently undergo extensive diagnostic workup which is usually unrevealing. These symptoms then resolve in the hospital as cannabinoid blood levels wane but recur when patients resume use of cannabinoids at discharge. The pathophysiology of CHS is not well understood. However, it is known that chronic marijuana use stimulates cannabinoid receptor type 1 (CB1) in the brain resulting in decreased peristalsis. It has been postulated that since CB1 lies closer to the thermoregulatory center in the hypothalamus, chronic hypothalamic stimulation of CB1 might be counteracted by hot water showering [7]. Alternative explanations in the literature include “cutaneous steal syndrome.” Chronic cannabinoid use causes CB1 receptor-mediated vasodilation of the gut. It has been theorized that the redistribution of blood from the gut to the cutaneous circulation with hot water bathing may be related to the temporary relief [4]. Diagnosis is often made in the presence of the cyclic nausea and vomiting, abdominal pain, and compulsive hot showers in the presence of chronic cannabis use. Based on the largest case series, diagnostic criteria of major and supporting features were proposed by Simonetto et al. [3, 8]. Based on these criteria, CHS may be diagnosed in a patient with long-term use of cannabinoid substances and any of the following:

- nausea and vomiting;
- abdominal pain;
- symptoms relieved by hot showers or baths;
- symptoms that resolve when cannabis is stopped;
- use of cannabinoid substance at least every week.

Other features found to be helpful include the following:

- younger than 50 years old;
- weight loss of at least 5 kg with symptoms;
- morning symptoms;
- normal bowel habits;
- negative laboratory, radiographic and endoscopic test results.

CHS is frequently overlooked. As reported in one case series, average time to diagnosis is 7 ± 4 visits to ED providers taking up to 9 years in cases before the diagnosis is made [5]. Testing should be geared towards excluding central nervous system, endocrine, metabolic, and gastrointestinal causes of nausea and vomiting after a careful history and physical examination. Cyclical Vomiting Syndrome (CVS), which commonly occurs in the setting of migraines and depression, should be considered in the differential diagnosis. However, long-term use of cannabinoid substances and compulsive hot water showering should point to CHS.

Management of CHS can be split into the treatment of acute hyperemetic phase and a chronic maintenance phase [6]. In the acute hyperemetic phase, inpatient care may be needed for supportive treatment. All classes of antiemetics may be used for intractable nausea and vomiting. Volume resuscitation is given for extracellular volume depletion which may be severe enough to cause renal insufficiency as in our case. Opioids for abdominal pain should be used with caution due to the possibility of potentiating emesis as a side effect. CHS patients may show esophagitis and gastritis; therefore, acid-suppressing medications may have a role in treatment but are by no means curative. These supportive treatments constitute the core of care in the acute hyperemetic phase, along with removal of the drug [6]. Care during the chronic maintenance phase involves prevention of relapse and patient education of the condition to help them understand cannabinoid substance use as the cause of their symptoms. Other measures to prevent relapse include cognitive behavioral therapy and drug rehabilitation programs [9].

In conclusion, CHS is a clinical entity that should be included in the differential diagnosis of nausea, vomiting, and abdominal pain in any patient with a history of cannabinoid use, especially when it is associated with obsessive hot bathing. Recognition of this syndrome is important in order to avoid costly and unnecessary clinical testing and to help the patient understand and break the cycle of drug use. A high index of suspicion is needed to make this diagnosis especially with synthetic cannabinoids that are not detected on conventional urine drug screens but are detected on gas chromatography mass spectrometry which are not routinely available [10].
